# Comparison of proton therapy techniques for treatment of the whole brain as a component of craniospinal radiation

**DOI:** 10.1186/1748-717X-8-289

**Published:** 2013-12-17

**Authors:** Jeffrey Dinh, Joshua Stoker, Rola H Georges, Narayan Sahoo, X Ronald Zhu, Smruti Rath, Anita Mahajan, David R Grosshans

**Affiliations:** 1 Departments of Radiation Oncology, The University of Texas M.D. Anderson Cancer Center, 1515 Holcombe Blvd., Unit 1150, Houston, TX 77030, USA; 2 Departments of Radiation Physics, Division of Radiation Oncology, The University of Texas M.D. Anderson Cancer Center, Houston, TX, USA

**Keywords:** Protons, CSI, Whole brain, Compensator, Passive scattering proton therapy, Spot scanning, Proton therapy, IMPT

## Abstract

**Background:**

For treatment of the entire cranium using passive scattering proton therapy (PSPT) compensators are often employed in order to reduce lens and cochlear exposure. We sought to assess the advantages and consequences of utilizing compensators for the treatment of the whole brain as a component of craniospinal radiation (CSI) with PSPT. Moreover, we evaluated the potential benefits of spot scanning beam delivery in comparison to PSPT.

**Methods:**

Planning computed tomography scans for 50 consecutive CSI patients were utilized to generate passive scattering proton therapy treatment plans with and without Lucite compensators (PSW and PSWO respectively). A subset of 10 patients was randomly chosen to generate scanning beam treatment plans for comparison. All plans were generated using an Eclipse treatment planning system and were prescribed to a dose of 36 Gy(RBE), delivered in 20 fractions, to the whole brain PTV. Plans were normalized to ensure equal whole brain target coverage. Dosimetric data was compiled and statistical analyses performed using a two-tailed Student’s t-test with Bonferroni corrections to account for multiple comparisons.

**Results:**

Whole brain target coverage was comparable between all methods. However, cribriform plate coverage was superior in PSWO plans in comparison to PSW (V95%; 92.9 ± 14 vs. 97.4 ± 5, p < 0.05). As predicted, PSWO plans had significantly higher lens exposure in comparison to PSW plans (max lens dose Gy(RBE): left; 24.8 ± 0.8 vs. 22.2 ± 0.7, p < 0.05, right; 25.2 ± 0.8 vs. 22.8 ± 0.7, p < 0.05). However, PSW plans demonstrated no significant cochlear sparing vs. PSWO (mean cochlea dose Gy(RBE): 36.4 ± 0.2 vs. 36.7 ± 0.1, p = NS). Moreover, dose homogeneity was inferior in PSW plans in comparison to PSWO plans as reflected by significant alterations in both whole brain and brainstem homogeneity index (HI) and inhomogeneity coefficient (IC). In comparison to both PSPT techniques, multi-field optimized intensity modulated (MFO-IMPT) spot scanning treatment plans displayed superior sparing of both lens and cochlea (max lens: 12.5 ± 0.6 and 12.9 ± 0.7 right and left respectively; mean cochlea 28.6 ± 0.5 and 27.4 ± 0.2), although heterogeneity within target volumes was comparable to PSW plans.

**Conclusions:**

For PSPT treatments, the addition of a compensator imparts little clinical advantage. In contrast, the incorporation of spot scanning technology as a component of CSI treatments, offers additional normal tissue sparing which is likely of clinical significance.

## Introduction

For treatment of the entire craniospinal axis, many practitioners consider proton therapy the radiation modality of choice [1,2]. The physical advantages of proton therapy for treatment of the spinal target are immediately apparent when comparisons of proton vs. photon spinal fields are made [3]. Additionally, benefits for particle therapy are seen when utilized for treatment of boost fields, such as sparing of the temporal lobes for patients with posterior fossa tumors [4].

The majority of proton treatments have been delivered using PSPT in which brass apertures are utilized to shape the lateral aspects of a large spread out proton beam [5]. The range of the proton beam, or distal edge, is controlled through the use of compensators. Compensators function to adjust the range of the beam across the target in order to conform the distal edge to the geometry of the target volume. For treatment of whole brain fields, as a component of CSI, compensators are commonly utilized in an attempt to reduce dose to cochleae and lenses [6]. However, the introduction of material into the beam path may inadvertently introduce dose heterogeneity, increase range uncertainty and in theory increase neutron contamination [7]. In contrast, with spot scanning proton therapy (SSPT), a pristine pencil beam is magnetically scanned lateral to the beam path and different energies are used to achieve the desired depth distributions [8-11].

In the current study we sought to evaluate the dosimetric consequences of utilizing compensators for PSPT in craniospinal radiation both for organs at risk and dose homogeneity, in a large cohort of brain tumor patients. We also sought to evaluate the potential benefits of spot scanning for such patients.

## Methods

Fifty consecutive brain tumor patients treated with craniospinal radiation were included. All patients were consented for and enrolled on prospective studies of proton therapy approved by the University of Texas MD Anderson Cancer Center institutional review board. Patient demographics and tumor histologies are presented in Table 1. Organs at risk (OARs) including the lens and cochlea along with target volumes (whole brain and cribriform plate) were contoured on the simulation computed tomography scan and each reviewed by a staff radiation oncologist. An Eclipse treatment planning system (Varian Medical Systems, Palo Alto, CA) was used for dose calculations and all plans generated using 2.5 mm slice spacing. For this retrospective study, for each patient PSPT had been previously planned and delivered using a compensator which was manually edited in order to spare both cochlea and lens OARs as much as possible, while maintaining target coverage. For the present study, clinical PSW plans were copied and PSWO plans retrospectively generated by deletion of the compensator and dose-recalculated with the same beam line. In order to facilitate comparison, both PSW and PSWO plans were generated for a prescription dose of 36 Gy(RBE) in 20 fractions for all patients. For PSPT, the clinical target volume (CTV) was used for planning according to standard of practice, as described previously [12]. Two posterior oblique beams were utilized both for PSW and PSWO plans, as posteriorly angled beams have been shown to contribute to sparing of the lens while allowing adequate coverage of the cribriform plate [13].

**Table 1:** **Patient characteristics (*n*** **= 50)**

**Number of patients**		
	Male	30
	Female	20
Histology		
	Medulloblastoma	23
	Germ cell tumor	11
	PNET	4
	ATRT	4
	Ependymoma	2
	Choroid plexus carcinoma	2
	Glioma	2
	Ganglioglioma	1
	Pinealoblastoma	1

*Abbreviations: PNET* primitive neuroectodermal tumor; *ATRT* atypical teratoid rhabdoid tumor.

A subset of 10 patients was subsequently chosen for planning with multi-field optimized intensity modulated proton therapy (MFO-IMPT) [14]. Because a robust optimization technique [15] is not currently available in our clinical treatment planning system, for IMPT planning, a planning target volume (PTV) was used for optimization, which included both setup and range uncertainties, in line with our current clinical practice. For cochleae, the planning organ at risk volume (PRV) was defined as a 5-mm expansion from the cochleae. The optimization volume was then defined as PTV minus PRV for cochleae. The spot spacing was 7 to 9 mm. The lateral field margin in the beams-eye-view was set equal to 8 mm, i.e., one spot was allowed to be outside the optimization volume [16]. A 1-cm width, dose-limiting ring peripheral to optimization volume was used to shape the dose gradient exterior to target, and to eliminate boundary hot spots. Lenses and cochleae were nominally constrained to 10 and 28 Gy, respectively. The optimization included the cribriform plate as an additional target volume to facilitate prescription dose coverage. A 6.7 cm thick range shifter was placed at the end of the nozzle to enable coverage of shallow target volume regions. The air gap was kept as small as possible to minimize the spot size and yet large enough to have the sufficient clearance for treatment delivery.

In all cases, treatment planning was performed by dosimetrists and medical physicists experienced with each modality. Qualitative and quantitative evaluations were conducted for each treatment plan generated. Dosimetric data were compiled including mean cochlear dose (left and right), maximum lens dose (left and right), maximum brainstem dose etc. To evaluate target coverage, V95% was evaluated for the whole brain as well as cribriform plate. To evaluate dose homogeneity we calculated both the homogeneity index (HI = D5/D95) as well as the inhomogeneity coefficient (IC = D5-D95/Dmean) [17,18]. For each index a lower value indicates superior dose homogeneity. Statistical significance was determined by a two-tailed t-test with Bonferroni corrections employed to account for multiple comparisons.

## Results

For the patient cohort investigated, the mean age at simulation was 18 years with a range of 2 to 65 years. Thirty-five patients were ≤18 years of age. Sixty percent of patients were male (Table 1). Forty six percent of patients were treated for medulloblastoma. The second most common indication was germ cell tumor followed by less common histologies (Table 1).

**Figure 1:**
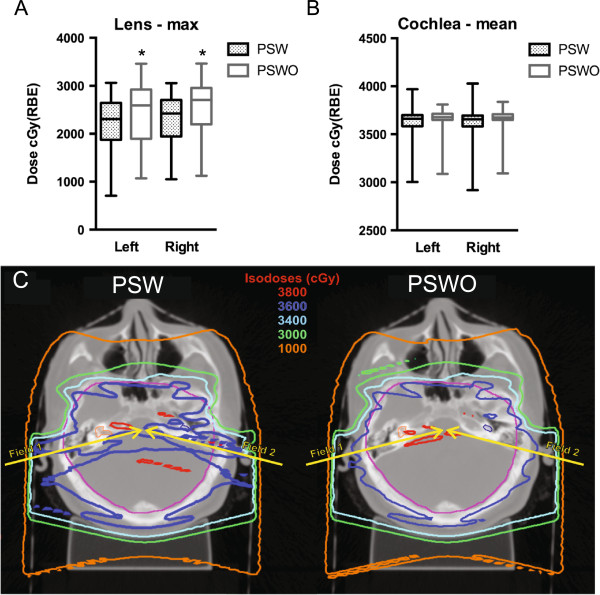
**Comparison of PSPT plans with and without compensators. (A)** Box-and-whisker plot of maximum lens dose, right and left, for PSW and PSWO plans. Vertical bars represent range and central bar median. **(B)** Box-and-whisker plot of mean cochlear dose, right and left, for PSW and PSWO plans. **(C)** Representative axial computed tomographic plans with and without compensators. Axial sections, at the level of the cochlea (highlighted in orange), demonstrate dose heterogeneity introduced by the compensator edge, extending through the brainstem. Yellow arrows depict the beam angles utilized. *significant vs. PSW, (p < 0.05), Student’s t-test with Bonferroni correction for multiple comparisons.

For both PSW and PSWO, whole brain target coverage was comparable (Table 2). However, the V95% for the cribriform plate was significantly higher for PSWO plans, an anatomical area which, if inadequately covered, may be associated with an increased risk of disease recurrence [19]. We next compared PSW and PSWO treatment plans in terms of OAR exposure. As expected, without the capacity for distal blocking offered by the addition of a compensator, PSWO plans had significantly higher maximum lens doses (Figure 1A). However, the addition of a compensator, offered no significant cochlear sparing (Figure 1B). Furthermore, qualitative review of plans suggested additional dose heterogeneity within the brainstem for PSW (Figure 1C). In order to quantitatively compare plan heterogeneity, we compared the homogeneity index (HI) and inhomogeneity coefficients (IC) for each plan type both for whole brain and brainstem. In comparison to plans generated with a compensator, PSWO plans were significantly more homogenous (Table 3). This was true both for the whole brain as well as for the brainstem where the magnitude of change was greater. This is presumably due to the close proximity of the brainstem and cochlea, where steep compensator edits would be expected to degrade plan homogeneity (Figure 1C).

**Table 2:** Comparison of target volume coverage

**Index**	**PSW (Gy(RBE) or %)**	**PSWO (Gy(RBE) or %)**
V95% - whole brain	98.5 ± 2.7	98.5 ± 2.8
D95 - whole brain	36.2 ± 0.9	36.1 ± 0.5
V95% - cribriform plate	92.9 ± 14	97.4 ± 5*

*Abbreviations*: *PSW* passive scatter with compensator; *PSWO* passive scatter without compensator; V95% = percentage of the target volume that receives at least 95% of the prescribed dose; D95 = dose volume histogram (DVH) curve dose representing 95% of volume of the target. Data presented as mean ± standard deviation; *significant vs. PSW (p < 0.05), Student’s t-test with Bonferroni correction for multiple comparisons.

**Table 3:** Dose heterogeneity

**Index**	**PSW (Gy(RBE) or %)**	**PSWO (Gy(RBE) or %)**
HI - whole brain	1.038 ± 0.01	1.031 ± 0.01*
IC - whole brain	0.036 ± 0.01	0.03 ± 0.01*
HI - brainstem	1.064 ± 0.06	1.028 ± 0.05*
IC - brainstem	0.06 ± 0.05	0.026 ± 0.04*

*Abbreviations*: *PSW* passive scatter with compensator; *PSWO* passive scatter without compensator; *HI* (homogeneity index) = D5/D95; *IC* (inhomogeneity coefficient) = D5% - D95%/Dmean; Data presented as mean ± standard deviation. *significant vs. PSW (p < 0.05), Student’s t-test with Bonferroni correction for multiple comparisons.

Based on the lack of cochlear sparing observed with both PSPT techniques. We next investigated the potential utility of spot scanning. Multi-field optimized IMPT plans, encompassing the cranium and cervical spine, were created utilizing one posterior (PA) and two anterior-oblique (AO) beams (left and right), all sharing a common isocenter. Employing a PA beam reduced thyroid dose and enabled coverage of the spine target inferior to the shoulder without reimaging, thus reducing the required number of isocenters inferiorly along the spine for most patients. For AO beams, the nominal beam angle was 75 degrees off the medial plane. This placement provided a beams eye view of much of the brain target, unencumbered by the dose-limiting cochlea. For the majority of plans, AO beams also included a 15-degree superior couch rotation, facilitating dose reduction to the eyes and lenses, while maintaining cribriform plate coverage. AO beams further ensured that target coverage near the dose sensitive lenses was not principally from the distal portion of the PA proton beam.

**Figure 2:**
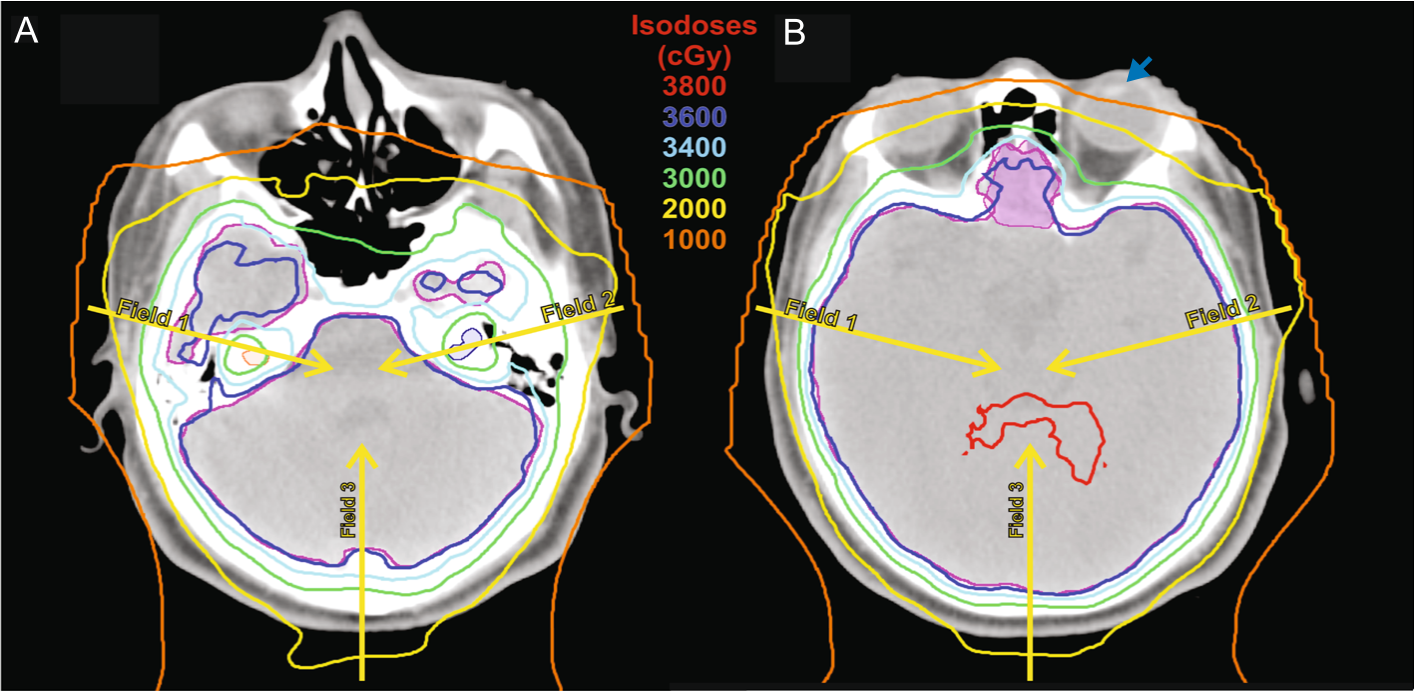
**Representative axial sections of a multi-field intensity modulated proton therapy plan.** Images demonstrate the capacity for **(A)** cochlear sparing (depicted in orange and blue color wash, right and left respectively) as well as **(B)** lens sparing (left lens highlighted by blue arrow) while maintaining coverage of the cribriform plate (opaque magenta). Yellow arrows depict the beam angles utilized.

IMPT plans displayed target coverage comparable to that of PSW plans (whole brain; V95% 99.8 ± 0.15, D95 36.5 ± 0.2 and cribriform plate; V95% 96.9 ± 2.4, D95 36.7 ± 0.3). In comparison to both PSW and PSWO techniques, IMPT plans demonstrated superior OAR sparing (Table 4, Figure 2A). However, utilizing the currently available optimization techniques, heterogeneity within the brain target was inferior compared to PSWO plans (whole brain; HI 1.053 ± 0.003, p < 0.05, Figure 2B) but similar when the brainstem was evaluated separately (brainstem; HI 1.04 ± 0.008, p = NS).

**Table 4:** Organs at risk

**Index**	**PSW (Gy(RBE))**	**PSWO (Gy(RBE))**	**IMPT (Gy(RBE))**
Left cochlea (mean)	36.4 ± 1.3	36.7 ± 1.0	28.6 ± 3.3^†^
Right cochlea (mean)	36.4 ± 1.4	36.7 ± 0.9	27.4 ± 1.5^†^
Left lens (max)	22.2 ± 5.5	24.8 ± 6.1*	12.5 ± 4.0^†^
Right lens (max)	22.8 ± 5.2	25.2 ± 5.9*	12.9 ± 5.0^†^
Brainstem (max)	39.3 ± 2.0	38.8 ± 2.0*	38.4 ± 0.5

*Abbreviations*: *PSW* passive scatter with compensator; *PSWO* passive scatter without compensator; *IMPT* intensity modulated proton therapy; Data presented as mean ± standard deviation; *significant vs. PSW, ^†^significant vs. PSW or PWO, (p < 0.05), Student’s t-test with Bonferroni correction for multiple comparisons.

## Discussion

Unnecessary radiation exposure to normal tissues, particularly in pediatric patients, is associated with increased risks of long-term adverse effects [20,21]. Lens and cochlear exposure in particular are associated with cataract formation and decreased hearing acuity respectively [22,23]. The current study, conducted in a large number of patients, supports the results of Jin et al. who also found that the addition of a compensator to PSPT increased heterogeneity [6]. This study adds additional information on the sparing, or lack thereof, of OARs as well as exploring potential benefits of IMPT. We found that the addition of compensators to whole brain treatments, as a component of CSI delivered with PSPT, offered modest lens sparing and little cochlear sparing at the expense of added heterogeneity. Moreover, cribriform plate coverage was superior in PSWO plans compared to PSW. Whole brain treatment plans generated using discrete spot scanning IMPT, displayed optimal target coverage along with superior sparing of lens and cochlea in comparison to either PSPT technique. However, dose heterogeneity was increased in IMPT plans.

Sensorineural hearing loss is common following brain irradiation. Especially in pediatric patients, diminished hearing may predispose to impaired communication skills resulting in diminished cognitive development and ultimately inferior quality of life. For children, treated with radiation alone, it has been suggested that cochlear doses be limited to less than 35 Gy in order to reduce the risk of ototoxicity [23]. A similar dose response is likely present in adult patients [24]. The addition of platinum based chemotherapy, as in the treatment of medulloblastoma, is expected to further increase the risk of cochlear damage [25,26]. In comparison to patients treated with photon techniques, including IMRT, published studies have demonstrated that patients treated to the craniospinal axis with PSPT have favorable hearing outcomes with low rates of high grade hearing loss [27-29]. These results highlight the clinical benefits of proton therapy and are likely due to cochlear sparing during the boost portion of therapy which is superior to photon techniques [2]. However, nearly 50% of patients did experience low-grade ototoxicity after PSPT based CSI, suggesting further room for improvement [27]. Thus, our finding that IMPT reduced cochlear doses compared to PSPT as part of whole brain treatment may have clinical significance.

In contrast to therapy-induced ototoxicity, which is largely irreversible, radiation-induced cataracts may be addressed surgically. However, clinical outcomes following lens replacement may be defined by the health of other remaining ocular structures [30]. Similar to otic structures, the exact dose response of the lens is complicated and influenced by both patient and radiation related factors such as fraction size and dose rate among others [31-33]. Regardless, additional sparing of both lens and other optic structures maybe expected to potentially avoid unnecessary surgical interventions. While we found that lens doses with PSWO plans were significantly higher than PSW plans. IMPT plans demonstrated the best lens sparing, while maintaining cribriform plate coverage. Based on studies of lens sparing during fractionated radiation therapy, the additional sparing offered by IMPT would be expected to reduce the incidence of cataract formation [34].

The current study did not include a comparison of photon based intensity modulated radiation therapy (IMRT). However, previous work has shown that IMRT plans have significantly higher dose heterogeneity in comparison to PSPT plans. However, of note the reported HI values are similar to those we recorded in MFO-IMPT planning [35]. Regardless, given the potential setup uncertainties which would be introduced by patient transfer between photon and proton treatment rooms, mixed modality CSI (IMRT brain and proton spine) is not clinically favored.

In our current clinical practice, we utilize PSPT without the routine use of compensators for treatment of the craniospinal axis. Many new proton centers will have the capacity for spot scanning therapy and some will exclusively employ this modality. The safe delivery of radiation to the entire craniospinal axis is technically challenging regardless of the radiation technique. While published work suggests that CSI delivered with PSPT is safe and efficacious [1,36], additional *in silico* and clinical studies will be necessary in order to implement CSI treatment using scanned beams. This is highlighted by the present study where scanned beam plans were found to be more inhomogeneous than PSWO plans. Further study including the adaptation of alternate optimizers, novel junctioning techniques etc., is expected to further improve dosimetric outcomes and to make CSI delivered with spot scanning a clinical reality. It is hoped that this will translate into further improvements in outcomes, including reduction of lens and cochlear toxicities.

## Abbreviations

AO: Anterior-oblique; ATRT: Atypical teratoid rhabdoid tumor; CSI: Craniospinal radiation; CTV: Clinical target volume; DVH: Dose volume histogram; HI: Homogeneity index; IC: Inhomogeneity coefficient; MFO-IMPT: Multi-field optimized intensity modulated; OARs: Organs at risk; PNET: Primitive neuroectodermal tumor; PRV: Planning organ at risk volume; PSPT: Passive scattering proton therapy; PSW: Passive scattering proton therapy with compensator; PSWO: Passive scattering proton therapy without compensator; PTV: Planning target volume; SSPT: Spot scanning proton therapy.

## Competing interests

The authors declare that they have no competing interests.

## Authors’ contributions

JD compiled and analyzed dosimetric data and drafted the manuscript. JS developed IMPT methodologies and treatment plans, compiled and analyzed dosimetric data and drafted the manuscript. RHG developed PSPT plans and compiled dosimetric data. NS conceived of the concept of the study and oversaw its completion. XRZ participated in the development of IMPT methodology and treatment planning. SR compiled and analyzed dosimetric data. AM conceived of the concept study and participated in its completion. DRG conceived of the study concept, participated in all aspects of its design and coordination and helped to draft the manuscript. All authors read and approved the final manuscript.
